# Gravity Data Fusion and Imaging of Geological Structures in the Red River Fault Zone and Adjacent Areas

**DOI:** 10.3390/s25041101

**Published:** 2025-02-12

**Authors:** Guiju Wu, Fei Yu, Hongbo Tan, Jiapei Wang, Weihua Liu

**Affiliations:** 1 Key Laboratory of Earthquake Geodesy, Institute of Seismology, CEA, Wuhan 430071, China; 13731310544@163.com (F.Y.); tanhongbo@hubdzj.gov.cn (H.T.); wangjiapei@hubdzj.gov.cn (J.W.); 2 School of Electric & Electronic Engineering, Wuhan Polytechnic University, Wuhan 430023, China; whliu2020@whpu.edu.cn

**Keywords:** Red River fault, Bouguer gravity anomaly, bilinear interpolation, gravity field model data, apparent density imaging

## Abstract

The geological structure in the Red River fault zone (RRF) and adjacent areas is complex. Due to the lack of high-precision gravity data in the study area, it is difficult to obtain the distribution of materials within the Earth’s crust. In this study, a gravity data-fused method is proposed. The Moho depth model data are utilized to construct the gravity anomaly trend, and the mapping relation between the gravity field model data and the measured gravity data is established. Using 934 high-precision measured gravity data as control points, the bilinear interpolation method is used to calculate high-precision grid data of the RRF. Finally, the apparent density inversion method is used to obtain clear crustal density images across the RRF. The experimental results show that the fuses data not only reflect the regional anomaly trend but also maintain the local anomaly information; the root-mean-square error of the fused data is less than 5% and the correlation coefficient is greater than 90%. Through an in-depth comparative analysis of density images, it is found that the low-density anomalous zones, with depths of ~20 km in the northern and southern sections of the RRF, are shallower than those in the middle. The data-fused method provides a new way to process geophysical data more efficiently.

## 1. Introduction

**Figure 1:**
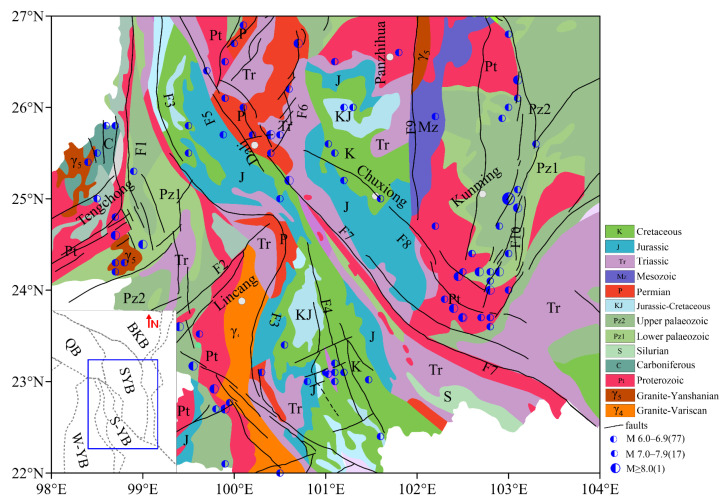
Schematic map of the geological structure (modified after [26,27]). F1: Nujiang fault; F2: Nantinghe fault; F3: Lantsang fault; F4: Wuliangshan fault; F5: Weixi–Weishan fault; F6: Binchuan–Yongsheng fault; F7: Red River fault; F8: Huanan–Chuxiong–Jianshui fault; F9: Anninghe fault; F10: Puduhe fault; F11: Xiaojiang fault; SYB: Sichuan–Yunnan block; BKB: Bayankala block; QB: Qiangtang block; S-YB: South Yunnan block; W-YB: West Yunnan block.

Large faults are known to be closely associated with destructive earthquakes, thereby attracting the attention of geologists and geophysicists [1,2]. A large strike-slip fault, the Red River fault (RRF), is located along the southwestern margin of the Sichuan–Yunnan block and adjacent to the southeastern margin of the Qinghai–Tibet Plateau. Since the Neogene period, the RRF has undergone a kinematic transformation, transitioning from an initial left-lateral strike-slip movement to a subsequent right-lateral strike-slip movement [3,4,5]. The middle segment, extending from Yuanjiang to Yuanyang, aligns in a north–northwest direction (60° N–70° W), forming a concave shape restraining a big bend and exhibiting thrust components [6,7,8]. In the northern and southern sections of the RRF, numerous strong M ≥ 6.0 earthquakes have occurred, but none have occurred in the middle section shown in Figure 1. These complex tectonic phenomena illustrate the intricate characteristics of the deep geophysical field surrounding the RRF. Due to the geographical constraints along and around this fault zone, high-precision geophysical data are lacking in this region. Therefore, obtaining high-precision geophysical data is of great significance for elucidating the crustal structure information from shallow to deep along the RRF, as well as for researching the conceiving and occurrence of strong earthquakes.

The results of tomographic imaging and the joint inversion of Rayleigh-wave dispersion and receiver functions have shown a low-velocity layer from the upper to middle and lower crust between Zhongdian and Xichang [9,10,11,12,13]; other studies have indicated that there is an obvious high-velocity anomaly in the lower crust beneath the central Sichuan–Yunnan block (SYB) [14,15,16]. The results of artificial seismic sounding profiles have revealed that the low-velocity anomaly in Zhongdian may have been caused by deep upwelling magma [17,18]. Li Yu et al. obtained Rayleigh velocity results for the SYB that showed an obvious low-velocity anomalous body in the middle and lower crust of the SYB, and the upper boundary of the low-velocity body was located at a depth of approximately 19 km [19]. Electromagnetic surveys showed two main low resistance zones at depths of 20–40 km, which may be a lower crustal “channel flow”; one of these zones runs through the SYB from NW to SE [20,21,22]. Previous gravity studies have focused on the activity and direction of the north and south sections of the RRF, respectively [23,24,25]. Due to the differences in the data and methods used by researchers, as well as the obtained differences in the geophysical characteristics, considerable controversies in understanding the distribution characteristics of deep structures in the SYB and its adjacent regions are still present.

In this study, the mapping relation between the gravity model data and the measured gravity data is obtained by taking the measured gravity data as the control point and the Moho surface fluctuation as the trend. The high-precision grid data in the study area were calculated via the bilinear interpolation method (BIM). Then, the apparent density method was used to identify the across-depth structural characteristics of the RRF. The seismogenic structural characteristics of strong earthquakes along the RRF and the formation mechanism of seismic gaps in the middle of the RRF were analyzed.

## 2. Material and Methods

### 2.1. Study Area

The study region spans the South Yunnan and Sichuan–Yunnan blocks from west to east (Figure 1). The main faults studied in this paper are oriented NW–SE, such as the Qujiang fault (F9), Red River fault (F7), Wuliangshan fault (F4), and Lantsang fault (F3); several faults are oriented N–S: the Nujiang fault (F1), Anninghe fault (F9), Puduhe fault (F10), and Xiaojiang fault (F11). The strata in the whole study region are mainly Jurassic, Triassic, Permian, and Cretaceous, accompanied by older Proterozoic, upper Paleozoic, and lower Paleozoic, and the intruded granites are Variscian and Yanshanian and are located to the west of F3. The strata along F7 from north to south are mainly Permian, Triassic, Jurassic, and Proterozoic. F7 marks the southwestern boundary of the SYB and extends southeast from Tibet to the South China Sea [28,29]. It is also a major intracontinental fault with right–lateral slip movements, fit for the South China block, which extrudes to the southeast [30,31,32,33]. Due to the collision and compression between the Indian Plate and the Eurasian Plate, the eastward movement of materials in the middle and lower crust is blocked by the rigid South China block, which leads to the thickening of the lower crust beneath the SYB [34]. But the crustal thickness on both sides of the RRF does not change abruptly; instead, it varies significantly along the RRF, with the northern and southern segments being thinner than the middle segment [35]. Strong earthquakes of M ≥ 6.0 all occurred in the northern and southern sections of F7, but none occurred in the middle section of F7. In other words, there are seismic gaps in the middle section of F7. Is this phenomenon caused by the clockwise rotation and overall motion of the SYB towards the SE due to the eastward flow of material from the Qinghai–Tibet Plateau? To answer this question, it is of great scientific significance and reference value to study the deep structural characteristics of this region for strong earthquake generation mechanisms and regional disaster prediction.

### 2.2. Bouguer Gravity Anomalies’ Data

**Figure 2:**
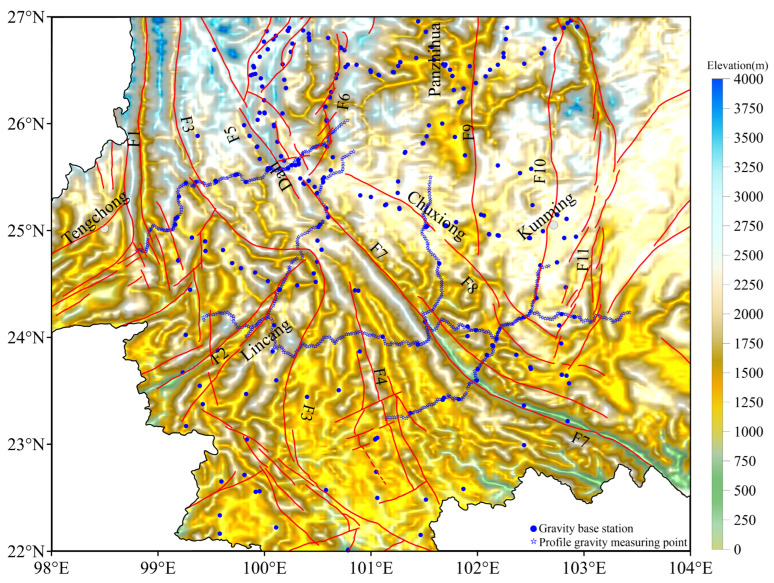
Topographic relief and distribution of observation stations map. The blue stars indicate the measured gravity profiles (709 points). The blue circles indicate the gravity base stations (225 points). Cities are marked with solid gray circles.

In this study, three kinds of data were utilized for gravity data fusion: gravity base stations’ data, gravity profiles’ data, and the Earth global gravity model EGM2008 [36]. As shown in Figure 2, there are 225 gravity base stations, and they are relatively evenly distributed throughout the study area (blue circles in Figure 2). A total of 5 gravity profiles were measured, with an average measuring point distance of approximately 2.5 km and a total of 709 (blue stars in Figure 2). These measuring points were measured using two gravimeters (CG–5/CG–6 Autograv^TM^, Scintrex, ON, Canada); the built-in software of the gravimeters was used for tide correction. A total of 5 data points were recorded at each gravity measuring point, and one data point was recorded per 60 s, with an accuracy of 5 × 10^−8^ m·s^−2^, and the average value of the 5 data points was the final measured value of the points. In the measurement process, the initial measuring point on the second day was the end measuring point on the previous day. The average distance of the gravity base stations is about 40 km, and the average distance between adjacent points of the measured gravity profiles is approximately 2.5 km, but the data coverage is too limited to provide a clear image of the physical distribution characteristics of the subsurface region.

**Figure 3:**
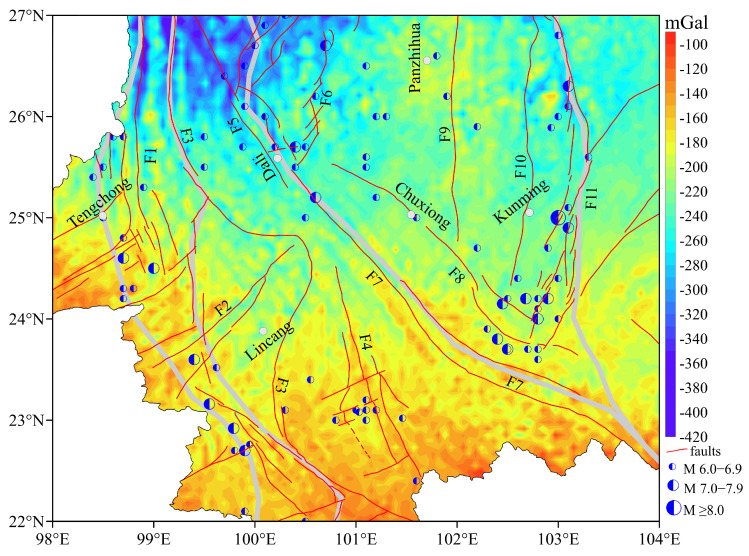
The BGAs of EGM2008 in the study area.

The Bouguer gravity anomaly (BGA) grids of the EGM2008 exhibit dramatic variations across the study, as shown in Figure 3. These anomalies were computed using the spherical harmonic coefficients of the EGM2008 and the density for the topographic correction is 2.67 g/cm^3^. They mainly reflect the relief of terrain and are consistent with the patterns of the topographic relief, and the density value used for topographic correction is not suitable for all regions. Furthermore, the grid data distance is averaged over 6.0 km × 6.0 km; the data resolution is too low to fully understand the crustal tectonic characteristics in the study area.

The coordinate systems utilized for the three kinds of gravity data are inconsistent, leading to differing longitude and latitude coordinates for each gravity data point. Furthermore, there are significant differences in elevation values even for the same points, which results in significant variations in the gravity data. In order to obtain more comprehensive, accurate, and reliable gravity anomaly data, it is necessary to unify these three kinds of gravity data into the same coordinate system. In this paper, the gravity profile data and the gravity base station data are selected as the control points to find the difference between the gravity model data and the measured data.

### 2.3. Incomplete Mapping Relation

The Moho surface undulation of CRUST1.0 is used to fit the unknown polynomials and the coefficients of their gravity profile [37]. The least square method is applied in this paper. The number of points is *n*; the multinomial (*m* − 1) formula is fitted as follows:

(1) $$f_{m}\left(x\right)=a_{1}+a_{2}x+a_{3}x^{2}+\cdots +a_{m}x^{m-1},$$

where *m* ≤ *n*. In the actual calculation process, *x* needs to be transformed in order to prevent operation overflow.

(2) $$\bar{x}=\sum_{i=1}^{n}x_{i}/N,$$

The form of the fitting polynomial is as follows:

(3) $$f_{m}\left(x\right)=a_{1}+a_{2}\left(x-\bar{x}\right)+a_{3}{\left(x-\bar{x}\right)}^{2}+\cdots +a_{m}{\left(x-\bar{x}\right)}^{m-1},$$

Then, the coefficients of the unknown polynomial (*a*_1_, *a*_2_, …) are determined. The incomplete mapping relation between the measured data and the gravity model data is as follows:

(4) $$G_{f}=G_{m}-0.308*h-f(h),$$

There, *G_m_* is the corrected the BGAs of the EGM2008 based on the measured gravity data and the GPS results from co-located observations [38]; *h* is the altitude difference between the elevation of the model digital terrain and the measured elevation at the same point, and *f*(*h*) is the approximate difference function between measured data and the gravity model data,

(5) $$f(h)\approx 0.00002679h^{4}-0.0003116h^{3}+0.01288h^{2}-1.6612h,$$

where the coefficients of the least square method in Formula (5) are fitted according to the Moho surface undulation. Based on the empirical formula of a previous study [39], the coefficients are modified. If the root-mean-square error (RMSE) is less than 5% and the correlation coefficient (CORREL) is greater than 90%, then the calculation ends.

(6) $$RMSE=\sqrt{\frac{\sum_{i=1}^{N}(G_{obsi}-G_{mi}{)}^{2}}{N}},$$

(7) $$CORREL=\frac{\sum_{i=1}^{N}\left(G_{obsi}-\bar{G_{obs}})*(G_{mi}-\bar{G_{m}}\right)}{\sqrt{\sum_{i=1}^{N}(G_{obsi}-\bar{G_{obs}}{)}^{2}*(G_{mi}-\bar{G_{m}}{)}^{2}}},$$

where *G_obs_* is the measured Bouguer gravity data, $\bar{G_{obs}}$ is the average value of the measured Bouguer gravity data, *G_mi_* is the Bouguer of gravity model data, and $\bar{G_{m}}$ is the average value of the Bouguer gravity model data. Finally, BIM is used to obtain high-precision grid data in the study area.

### 2.4. Bilinear Interpolation Method

**Figure 4:**
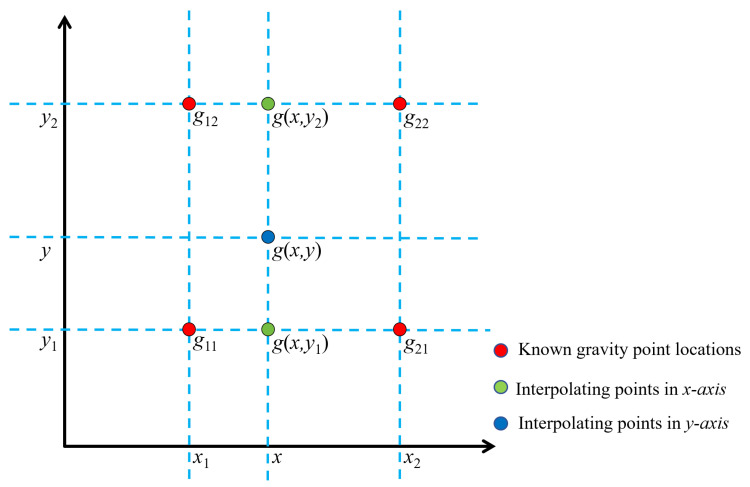
Diagram of the bilinear interpolation method.

The BIM is a two-dimensional application of linear interpolation [40,41]. This method calculates the pixel value of the target pixel by considering the four nearest neighboring pixels surrounding it and using linear weights to perform a weighted average on them. It not only can provide relatively smooth reconstruction results but also avoid abrupt changes in pixel values, making the interpolated image smoother and more natural. In this paper, the value of the output BGAs is the weighted average of the input BGAs in the nearest 2 × 2 neighborhoods. Firstly, a 2.5 km × 2.5 km grid is built, and the corrected BGAs data and the BGAs of measured data are filled into the grid; meanwhile, the BGAs of the measured data are used as the control points. The BGA values of the vertex coordinates of the square region and their coordinates are *g*_11_(*x*_1_, *y*_1_), *g*_21_(*x*_2_, *y*_1_), *g*_12_(*x*_1_, *y*_2_), and *g*_22_(*x*_2_, *y*_2_), as shown in Figure 4.

The *g*(*x*, *y*) of any point in the square can be obtained using BIM. *g*(*x*, *y*_1_) is obtained by interpolating points *g*_11_ and *g*_21_ in the *x* direction,

(8) $$g(x,y_{1})=\frac{x_{2}-x}{x_{2}-x_{1}}g_{11}+\frac{x-x_{1}}{x_{2}-x_{1}}g_{21},$$

*g*(*x*, *y*_2_) is obtained by interpolating points *g*_12_ and *g*_22_ in the *x* direction,

(9) $$g(x,y_{2})=\frac{x_{2}-x}{x_{2}-x_{1}}g_{12}+\frac{x-x_{1}}{x_{2}-x_{1}}g_{22},$$

and *g*(*x*, *y*) is obtained by interpolating points *g*(*x*, *y*_1_) and *g*(*x*, *y*_2_) in the *y* direction,

(10) $$g(x,y)=\frac{y_{2}-y}{y_{2}-y_{1}}g(x,y_{1})+\frac{y-y_{1}}{y_{2}-y_{1}}g(x,y_{2}),$$

Formulas (8) and (9) are substituted into Formula (10). Finally, *g*(*x*, *y*) is as follows:

(11) $$g(x,y)=\frac{g_{11}}{\left(x_{2}-x_{1})(y_{2}-y_{1}\right)}(x_{2}-x)(y_{2}-y)+\frac{g_{21}}{\left(x_{2}-x_{1})(y_{2}-y_{1}\right)}(x-x_{1})(y_{2}-y)\;+\frac{g_{12}}{\left(x_{2}-x_{1})(y_{2}-y_{1}\right)}(x_{2}-x)(y-y_{1})+\frac{g_{22}}{\left(x_{2}-x_{1})(y_{2}-y_{1}\right)}(x-x_{1})(y-y_{1}),$$

### 2.5. Apparent Density Imaging Methodology

The apparent density imaging is a method of dividing the underground medium into a multi-layer and multi-block density model, establishing the objective function in the spatial domain, and solving it with the optimization inversion method so as to obtain the heterogeneous distribution of the density of the underground medium with depth. In this study, suppose there are an observational gravity anomaly profile *d* = {*d*_1_, *d*_2_, …, *d_M_*} and an unknown residual density model, *m* = {*m*_1_, *m*_2_, …, *m_N_*}, which are related by

(12) Gm=d, 

where $G\in R^{N\times M}$ is a sensitivity matrix called the kernel matrix, and the element $G_{ij}$ represents the gravity anomaly weight generated via the *j*th density cell at the *i*th data point.

In general, the number of residual density cells, *N*, is much larger than the number of observational gravity anomaly data points, *M*, and Equation (12) cannot be solved directly [42]. The inverse problem (density imaging) can be transformed into an optimization problem by introducing some a priori constraints and using regularization. We follow the Li and Oldenburg scheme in this study [43], and the global objective functions are given by

(13) $$\Phi \left(m\right)={\left\|W_{d}\left(d-Gm_{0}\right)\right\|}^{2}+\mu {\left\|W_{m}\left(m-m_{0}\right)\right\|}^{2}-2\lambda \sum_{j=1}^{N}ln\frac{m_{j}}{m_{max}},$$

The first term of Equation (13) is the data misfit function, $m_{0}$ is an initial reference density model, and $W_{d}$ is a weighting matrix of the data [44].

(14) $$W_{d}=diag\left\{1/\sigma_{1}^{2},\right.1/\sigma_{2}^{2},\cdots ,\left.1/\sigma_{M}^{2}\right\},$$

$\sigma_{i}$ is the standard deviation in the *i*th data point.

The second term of Equation (13) represents the objective function of the model, and μ is fixed regularization parameter that can be controlled via the objective function and the data misfit function. $W_{m}$ is the model weighting matrix, which consists of four matrices, and the form of any of the four matrices is as follows:

(15) $$W_{mi}=\alpha_{i}S_{i}D_{i}Z,$$

where i=s,x,y,z. $\alpha_{i}$ is a coefficient that gives relative weighting to each matrix. $\alpha_{x}=\alpha_{y}=\alpha_{z}=1$ and $\alpha_{s}=0.05$ in this study. Ss is a diagonal matrix with weighting for each cell, and Sx, Sy, and Sz are diagonal matrices with weighting for each interface in their corresponding direction; Ds is a diagonal matrix with $\sqrt{dxdydz}$ on its diagonal, and Dx, Dy, and Dz are derivative operators for the respective direction. The depth weighting function *Z* is given by

(16) $$Z=\frac{1}{{\left(z_{0}+z\right)}^{\beta /2}}$$

*Z* is the depth of the residual density cell center, and $z_{0}=1$ and β=3 in the study [38,43].

The third term of Equation (13) is an additional regularization term that imposes the positivity of the tail effect using a logarithmic barrier method. *λ* is the logarithmic constant, $m_{j}$ is the density of the *j*th cell, and $m_{max}$ is the maximum possible residual density.

The optimal residual density model is solved through iteration until two stopping criteria are met: if *λ* is sufficiently small or nearly zero, the logarithmic term has almost no contribution to Equation (13), and the change in the objective function in the previous iteration is less than 1%. Finally, the apparent density imaging method is used to invert the data from the five gravity profiles. In the paper, the constraint range for apparent density is from −1 g/cm^3^ to 1 g/cm^3^, based on the results of artificial seismic wave velocity measurements [34].

## 3. Results

### 3.1. The Result of Data Fusion

**Figure 5:**
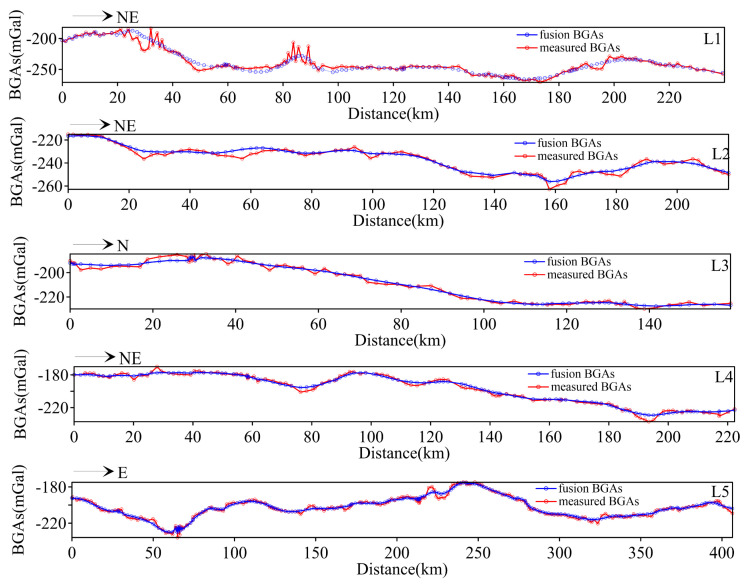
A comparison of the data before and after fusion along the measured profiles.

Figure 5 displays a comparison of the BGA data from the actually measured profiles at the same data locations and the fused data. As can be seen from Figure 5, along profile L1, profile L2 and, profile L4, the BGAs gradually decrease from the northwest to the southeast, and the overall trend remains consistent across all points. Along the NS profile L3, the BGAs also gradually decrease from south to north. Along profile 5, the overall trend of the BGAs exhibits a descending–ascending–descending pattern. The fused data appear smoother and denser, not only reflecting regional anomaly trends but also preserving local anomaly information. In the calculation, the RMSE is 2.96, and the CORREL is 0.91. This demonstrates the correctness of the mapping relationship established, providing more accurate data for crustal structure analysis.

**Figure 6:**
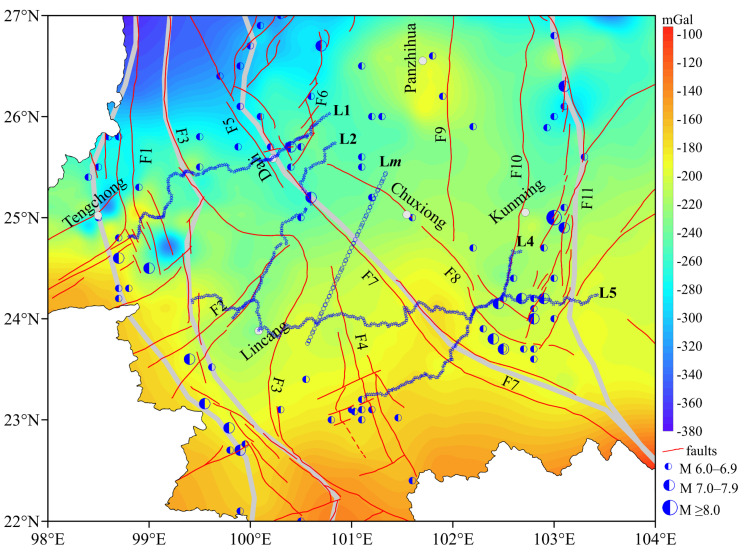
The BGAs after fusion in the study area.

The overall fused data of BGAs in the study area are shown in Figure 6. The BGAs of high-precision grid data are averaged over 2.5 km × 2.5 km. The entire BGAs in the study area are negative and decrease gradually from south to north. Compared to Figure 3, the fused data not only retain useful information but also eliminate the influence of terrain. The amplitude is −100–−380 mGal. M ≥ 6.0 earthquakes are usually distributed in the transition zone of the BGAs, and the faults in the regions are also developed. Upon comparing the stratigraphic and seismic distribution (Figure 1) with the results of fused BGAs (Figure 6), it is found that strong earthquakes often correspond to regions with scattered stratigraphic formations or stratigraphic boundary zones. These regions also coincide with the transition zones of BGAs, especially along the southern segment of F1, F4, the northern segment of F7, and F11.

In order to analyze the crustal density structure characteristics of the RRF, five gravity profiles crossing it are analyzed. The BGAs is a comprehensive reaction of underground materials. The BGAs of four measured gravity profiles (L1, L2, L4, and L5) and one extractive profile (L*m*) were analyzed, and then the apparent density method was used to obtain the across-depth structural characteristics of the RRF, as shown in Figure 6.

### 3.2. The Result of Density Imaging

**Figure 7:**
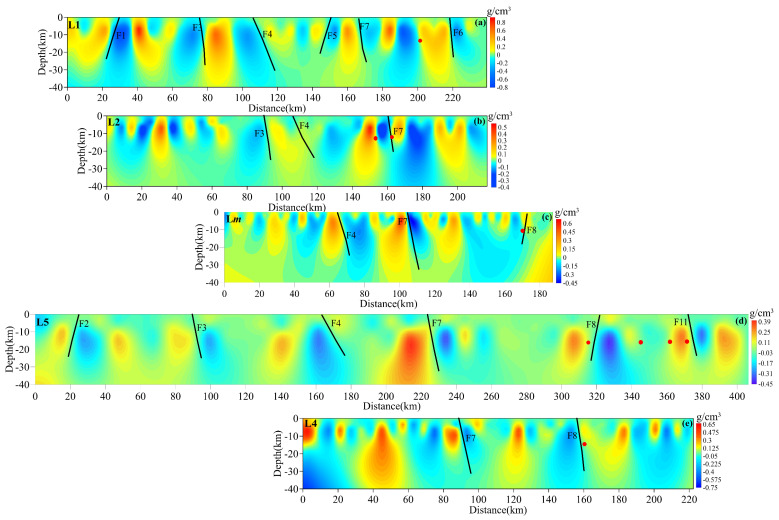
Density contrast results along five profiles beneath crossing the RRF from north to south. Subfigures (**a**–**e**) sequentially represent the density contrast results for L1, L2, L*m*, L5, and L4. The black lines indicate faults. The red circles indicate the projected locations of earthquakes with M ≥ 6 that occurred along the profiles.

To accurately reveal the structural anomalies in the study region, the five gravity profiles were calculated in more detail using the apparent density imaging method, as shown in Figure 7. Profile L1 spans the F1, F3, F4, F5, F7, and F6 faults from southwest to northeast. The values of residual density contrasts are alternately positive and negative along the profile (Figure 7a). When faults are projected onto L1, they intersect at approximately 30, 75, 110, 150, 180, and 220 km from the beginning (southwest of profile L1) of the x-axis along profile L1 in the NE direction. These faults all correspond to the negative anomalous area of the density contrasts. The dip angles of these anomalies are very steep, with angles > 50° (Table 1). Moreover, the negative anomalous areas extend downwards to a depth greater than 20 km, and the anomalous areas even penetrate downwards to a depth greater than 35 km at 30, 75, and 110 km in profile L1.

Profile L2 spans the F2, F3, F4, F7, and F6 faults from southwest to northeast, and the southwestern part of profile L2 is distributed along F2. The values of density contrast along profile L2 are mainly negative, except for two positive anomalous areas at approximately 30 and 150 km (Figure 7b). F2, F3, F4, F7, and F6 are projected onto the gravity profile L2 along the x-axis. These faults are located at ~95 and 165 km, respectively. Anomalous areas close to each other near 180 km along the x-axis intersect at a depth of ~15 km. In other words, the surfaces of F6 and F7 are close together, with dip angles > 50° (Table 1), and the large range of negative anomalous areas penetrates to >40 km in depth.

Profile L*m* spans the F4, F7, and F8 faults from southwest to northeast. The values of the density contrast along profile L2 are mainly alternating positive–negative distributions, especially a negative anomalous area at approximately 140 and 170 km (Figure 7c). Four negative anomalous areas are observed at approximately 50, 80, 110, and 150 km from the beginning (west of profile L*m*) of the x-axis, with depths greater than 30 km along the profile. F4, F7, and F8 are projected onto gravity profile L*m* along the x-axis, and these faults correspond to the three anomalies from west to east at 70, 110, and 165 km, respectively. The dip angles of the three anomalies are steep. Between 140 km and 170 km, the negative anomaly penetrates to >35 km in depth. It can be inferred that this negative anomaly corresponds to the southern boundary of SYB, and on either side of it, crustal structures are positive density contrasts.

Profile L5 is the longest with a distance > 400 km; it is in the W–E direction and spans F2, F3, F4, F7, the southern Sichuan–Yunnan block (SYB), and F10 from west to east. The density contrast imaging map is shown in Figure 7d. Six negative anomalies are observed at approximately 30, 100, 160, 240, 320, and 380 km from the beginning (east of profile L5) of the x-axis, with depths greater than 20 km along the profile. F2, F3, F4, F7, F8, and F11 are projected onto gravity profile L5 along the x-axis, and these faults correspond to the six anomalies from west to east. The dip angles of the six anomalies are steep, and on either side of the F11 crustal structures are positive density contrasts.

Using the same method, two negative anomalous areas are inferred at 90 and 150 km from the beginning (southwest of profile L4) of the x-axis, with depths greater than 20 km along profile L4 (Figure 7e). Another negative anomaly is also indicated at approximately 70 km along the x-axis; it converges with the negative anomaly at 90 km on the x-axis at a depth of approximately 35 km. It can be inferred that this negative anomaly corresponds to F7, and F7 is wider. The dip angles of the three negative anomalies are >70° (Table 1). Two positive anomalous areas are located between 60 and 110 km, with depths > 35 km. On two sides of F8, positive anomalies with depths > 35 km are also present. One negative anomalous area is inferred between 190 km and 210 km, with depths greater than 35 km along profile L4. In terms of geographical location, this area is mainly distributed along F10.

In this study area, the density contrasts are within the range of plus or minus 0.8 g/cm^3^. The major faults are located in the negative density contrast areas along the five profiles, with dip angles > 50°. The negative apparent density areas all penetrate to >20 km in depth. According to the records of the China Earthquake Networks Center, the focal depths of strong earthquakes with M ≥ 6 within the study area are generally around 15 km. When strong earthquakes along each profile are projected onto the respective profiles, they are generally located in the zones where the density anomalies transition from positive to negative, as illustrated in Figure 7. Furthermore, in comparison to the geological distribution depicted in Figure 1, these specific locations also align with stratigraphic boundaries.

## 4. Discussion

The high-precision BGA data in the Red River fault zone and adjacent areas were obtained. The density contrasts beneath the RRF were inversed. Therefore, we use the results of high-resolution data in space to discuss the following issues: (1) the accuracy of the fused data; (2) the characteristics of the RRF; and (3) the implications of the findings along the RRF.

### 4.1. The Accuracy of the Fused Data

The BGAs represent the superposition of the effect of both deep and shallow anomaly signatures [45,46]. The separation of the regional component (the Moho surface fluctuating trend effect) from the BGAs is essential for studying the near-surface effect above Moho [47]. The complete BGAs of EGM2008 are usually obtained through mathematical methods (such as spherical or ellipsoidal harmonics), combined with satellite observations, calculations, and processing. These data are limited by model assumptions, algorithm accuracy, data resolution, and other factors, which may not fully reflect the real situation of the Earth’s gravity field.

**Figure 8:**
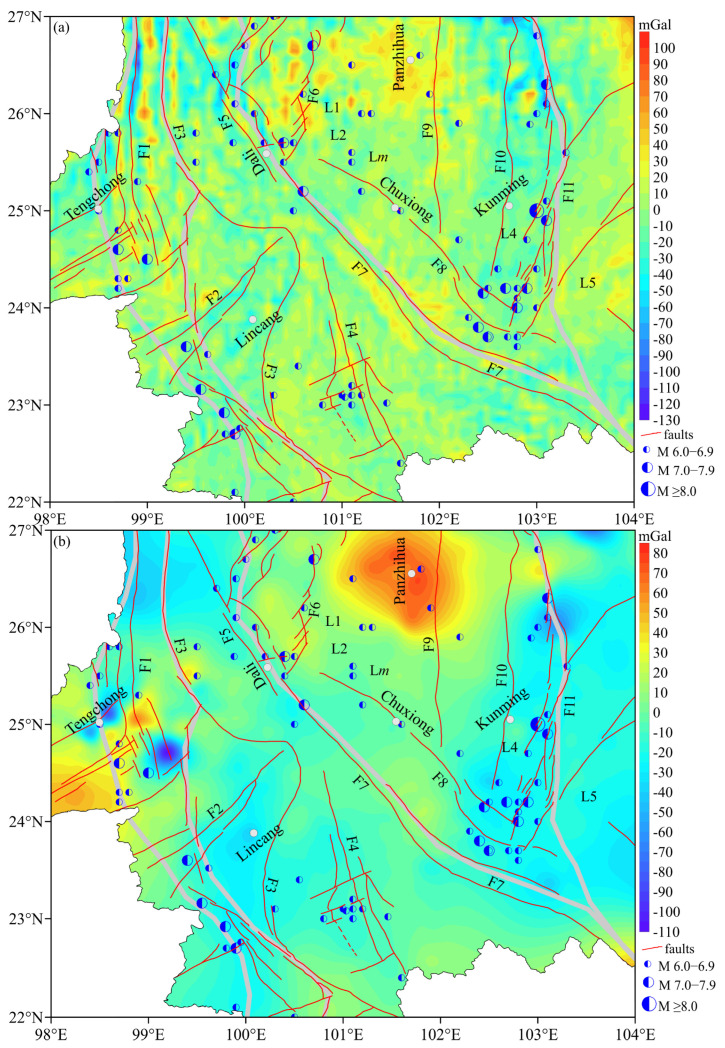
The residual BGAs of the research region, obtained by subtracting the regional values. (**a**) The residual BGAs of EGM2008; (**b**) the residual BGAs of the fused BGAs.

Figure 8a shows the residual BGA results of EGM2008 after the Moho surface fluctuating trend effect is removed. Compared with the terrain shown in Figure 2, the region with high altitude corresponds to the positive high anomaly region, and the region with low altitude corresponds to the negative low anomaly region, the residual BGAs reflect the characteristics of relief changes in the terrain. And the positive and negative regions of the residual BGAs are scattered.

Figure 8b shows the residual BGAs results of the fused BGAs. The faults are mainly distributed along the high–low gradient transition zone of the residual BGAs. At the same time, M ≥ 6.0 earthquakes usually occur in the negative abnormal areas. The block boundaries are also mainly distributed along the high–low gradient transition zone, and the residual BGAs along the block boundaries are mainly negative. Compared with the geology stratum shown in Figure 1, the high–low gradient transition zones of the residual BGAs also align with stratigraphic boundaries. Both the northern and southern sections of the RRF exhibit pronounced high–low gradients of the residual BGAs, whereas the middle section displays irregular distortions with a relatively low variation. This phenomenon is relatively consistent with other research findings [48,49]. These results demonstrate significant variations in rock density within the crust along the RRF, indicating a complex deep tectonic environment.

The residual BGAs are essentially a reflection of the uneven distribution of matter density inside the earth. By analyzing the distribution characteristics of residual BGAs, we can infer the strike, dip, and dip parameters of geological structures. The fused data in the research area not only reflect the regional anomaly feature but also retain the local anomaly information.

### 4.2. Characterizing Crossing the RRF

From north to south along F4 and F7, the apparent densities at depths ≤ 20 km are low and complex. The apparent densities are high-density anomalies on both sides of F4 and F7, and the anomalies, all penetrate downwards to greater than 20 km in depth. The locations and shapes of the low-density anomalous zones along F7 correspond to those of the low-speed zones [50,51,52,53,54]. The apparent densities along F7 at depths greater than 30 km are high–low–high from north to south.

In the northern section of F7, profiles L1 and L2 span this section, the low-density anomalous zones extend downwards to a depth of ~20 km, and the high-density anomalous zones on the western and eastern sides of F7 also penetrate to a depth of ~20 km, as shown in Figure 7a,b. The high-density anomalous zones mainly correspond to the Permian and Triassic. The low-density anomalous zones are located at the junction of the two geological strata. However, M ≥ 6.0 earthquakes often occur in areas with low BGAs near high-low gradient transition zone, especially earthquakes with M ≥ 7(Figure 8b).

In the middle section of F7, profiles L*m* and L5 span the section, and the low-density anomalous zones extend downwards to depths ≥ 35 km, as shown in Figure 7c,d, but the downward extension depth of the high-density anomalous zone to the west of F7 is greater than 40 km. In particular, the low-density anomalous zone is obvious at approximately 35 km (in profile L5) along the x-axis, and it extends from the surface to the middle and lower crust, as shown in Figure 7d. The low-density anomalous zones are mainly located at the junction of Proterozoic and Triassic strata, and east of F7, the apparent densities are mainly low-density anomalies corresponding to Jurassic strata. The distribution of earthquake epicenters shows that there have been no earthquakes with M ≥ 6.0 in the middle segment of the RRF, and even earthquakes with M ≥ 5.0 are rare.

In the southern section of F7, profile L4 spans the section, and the downward extension depth of the low-density anomalous zone is less than 30 km, as shown in Figure 7e. The low-density anomalous zone is consistent with the low-resistivity zone of magnetotelluric sounding results [20,21]. M ≥ 6.0 earthquakes often occur on the east side of F7 at high densities, which correspond to Proterozoic strata.

The low apparent densities beneath F7 are mainly caused by a deep and large fault cutting through the crust. However, the low apparent densities on the east side of F7 in the middle are higher than those in its northern and southern sections, while the high-density anomaly zone in the middle section of F7 extends downwards to deeper than that in the northern and southern sections. Along F7, M ≥ 6.0 earthquakes usually occur in areas with older strata, where the apparent densities are high-low gradient transition zone.

### 4.3. Causes of the Seismic Gap in the Middle of the RRF

Since the records began, strong M ≥ 6.0 earthquakes have all occurred in the northern and southern sections of F7, but none have occurred in the middle section of F7. In other words, there is a seismic gap in the middle section of F7, especially between Nanjian and Mojiang. That is, the crustal material along F7 is different in the north, middle, and south sections. The results of a geodetic survey indicate that the middle segment of the RRF exhibits a higher locking degree, with weaker strain compared to its northern and southern segments [55,56,57].

**Figure 9:**
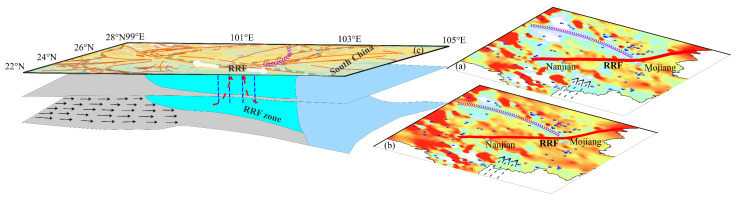
A simplified diagram of material distribution and crustal material flow along the RRF. (**a**) The reflection ratio of the residual BGAs; (**b**) the reflection ratio of the fused BGAs. The white lines with arrows indicate deep material flow in the middle of the RRF; (**c**) a cartoon diagram of crustal material flow along the RRF. The black lines with arrows mark the eastward-moving material. The red lines with arrows mark the upwelling mantle material, and the area with blue wavy lines indicates a high-density area. The purple lines with arrows mark indicate lower crust flow [14].

The crustal material signal reflection ratios along the RRF show that the reflectance ratios are mainly high in the middle of RRF and low in the north and south, as shown in Figure 9a. The reflection ratios along the lower crustal are low, especially in the area above the Moho shown in Figure 9a. And Figure 9b shows that the area of the reflection ratios in the middle of F7 are large and exhibit the effects of Moho fluctuations. The results of the average shear wave velocity (*Vs*) indicate that, in the depth range of 5–20 km, the values of Vs in the middle section of F7 are higher than that in the north and south [58,59,60] and lower at depths greater than 25 km. The high-density area corresponds to the high-speed belt. Low-density areas can lead to energy and material accumulation. However, the shallow to lower crust in the middle of F7 is dominated by high densities, which do not easily accumulate crustal material or energy. Research findings concerning the electrical structural characteristics of the crust indicate the presence of significant high conductors in the northern and southern segments of RRF [20,61,62]. Meanwhile, the reflection ratios are high in the middle of F7, as shown in Figure 9b. The eastward material flow of the Qinghai–Tibet Plateau in the crust is prevented by the SYB and moves northwards or southwards along F7, as shown in Figure 9c. Moreover, the crustal material is high-density in the middle of F7, where the material cannot accumulate. These results lead us to conclude that strong earthquakes do not often occur in areas with high densities.

In our calculated variations in density contrast, the overall trend of the variations in density contrast is large in the northern and southern sections of F7 and is small in the middle section of F7. It is interesting that the places where the strong earthquakes occurred agree with the area where the variations in density contrast are large along F7. Although M ≥ 6.0 earthquakes occurred frequently in the southern and northern sections of F7, as shown in Figure 1, and their focal depths were shallow, generally lower than 15 km, this phenomenon still does not affect the rate of densities’ variation. In other words, the amplitude of the density variation is the greatest in the middle of F7. In previous studies, there was a channelized weak zone across F7, which is still under debate [51,63,64,65,66,67]. Some researchers have proposed that a channelized weak zone continues at depths of 20–40 km in the southern direction across F7 and extends to northern Vietnam. Others indicated that the channelized weak zone approximately parallels F7. In terms of the obvious variation in densities, the small variation in the southern part of F7 may be due to the dipping barrier. This phenomenon seems to contradict the explanation that there exists a thick channelized weak zone parallel to F7. The eastward-moving material in the southern part of F7 and the south-eastward crustal flow material converge at the southern end of F7, which results in material accumulation.

The phenomenon is similar in the northern part of F7 that reaches the eastern Himalayas; the phenomenon may be caused by the high-density body formed by the blocking of the flow material in the crust, as shown in Figure 9. Previous results have shown that the crustal material in the north–central SYB is rigid, which blocks the eastward flow of material from the Qinghai–Tibet Plateau [68]. The results of the apparent densities along the northern section of F7 show that they are high. Moreover, the amplitude of the variation in gravity is large, which matches the above research. Other findings have shown that an ascending mantle material produced many intrusions, which were emplaced both inside and outside F7 [69] and may have formed soft and low apparent densities in the crust, especially in the middle section of F7 and its adjacent area. And some research results also indicate that, due to crustal movements and stress effects, the crust in the middle segment of the RRF may have already closed or become locked, forming a localized area of continuous deformation in the NW–SE direction [70,71]. Therefore, we suggest that another reason for the seismic gap in the middle of F7 is the migration of upwelling mantle material to the northern and southern sides of F7.

## 5. Conclusions

The high resolution of apparent density spatial images reveals well-resolved crustal structures beneath the Red River fault and its adjacent region, and continuous temporal gravity variation maps show obvious differences in the northern, middle, and southern F7. This study provides high-accuracy BGA grid data and important insights into the causes of strong earthquake gaps in this region. The major findings of this study can be summarized as follows:

1. The BGAs of high-precision grid data of 2.5 km × 2.5 km are obtained. The RMSE is about 2.96, and the CORREL is about 0.91. The fused data in the research area not only reflect the regional anomaly feature but also retain the local anomaly information.
2. The low-density anomalous zones in the northern and southern sections of F7 are shallower than those in the middle. The high-low gradient transition zones of densities are also located at the junction of geological strata and extend downwards to a depth of ~20 km, where M ≥ 6.0 earthquakes often occur, especially earthquakes of M ≥ 7.
3. The reflection ratios in the middle of F7 are higher than in the north and south of F7. The migration of upwelling asthenospheric material to the northern and southern sides of F7 cannot accumulate material and energy in the middle of F7, resulting in the existence of a strong earthquake gap.

## Figures and Tables

**Table 1:** Major fault information.

Fault Name	Proneness	Dip Angle	Fault Strike	Fault Property
Nujiang fault (F1)	W	~70°	NS	Strike−slip reverse fault
Nantinghe fault (F2)	SW	80~90°	NE	Right−lateral strike−slip fault
Lantsang fault (F3)	SW	~52°	N30°W	Right−lateral strike−slip fault
Wuliangshan fault (F4)	SE	60~70°	NNE	Right−lateral strike−slip fault
Weixi–Weishan fault (F5)	SW	~70°	NNW	Right−lateral strike−slip fault
Binchuan–Yongsheng fault (F6)	W	>50°	Nearly NS	Left−lateral strike−slip, normal fault
Northern Red River fault (F7)	NE	60~85°	N70°W	Right−lateral strike−slip, dip−slip reverse fault
Southern Red River fault (F7)	NE	60~85°	N45°W	Right−lateral strike−slip, dip−slip normal fault
Western Qujiang fault (F8)	NW	50~75°	N60°W	Right−lateral strike−slip, dip−slip reverse fault
Eastern Qujiang fault (F8)	NW	60~80°	N60°W	Right−lateral strike−slip, dip−slip reverse fault
Anninghe fault (F9)	E	70~80°	NS	Left−lateral strike−slip
Puduhe fault (F10)	W	70~80°	NS	Left−lateral reverse strike−slip
Xiaojiang fault (F11)	E	70~80°	NS	Left−lateral strike−slip, dip−slip normal fault

## Data Availability

The seismic event catalog is available through CENC (http://www.ceic.ac.cn/history) and accessed on 8 June 2024. The complete EGM2008 Bouguer gravity anomalies data were extracted from the official Earth Gravitational Model (EGM2008) on 18 March 2020 released by the National Geospatial Intelligence Agency (NGA) (http://bgi.obs-mip.fr/data-products/outils/egm2008-anomaly-maps-visualization/). These data accessed on 1 January 2020.
